# Abnormal resting-state functional connectivity underlies cognitive and clinical symptoms in patients with schizophrenia

**DOI:** 10.3389/fnhum.2023.1077923

**Published:** 2023-02-15

**Authors:** Yingxin Jia, Namasvi Jariwala, Leighton B. N. Hinkley, Srikantan Nagarajan, Karuna Subramaniam

**Affiliations:** ^1^Department of Radiology and Biomedical Imaging, University of California, San Francisco, San Francisco, CA, United States; ^2^Department of Psychiatry, University of California, San Francisco, San Francisco, CA, United States

**Keywords:** schizophrenia, resting state, magnetoencephalography, cognitive symptoms, psychotic symptoms

## Abstract

**Introduction:**

The cognitive and psychotic symptoms in patients with schizophrenia (SZ) are thought to result from disrupted brain network connectivity.

**Methods:**

We capitalize on the high spatiotemporal resolution of magnetoencephalography imaging (MEG) to record spontaneous neuronal activity in resting state networks in 21 SZ compared with 21 healthy controls (HC).

**Results:**

We found that SZ showed significant global disrupted functional connectivity in delta-theta (2–8 Hz), alpha (8–12 Hz), and beta (12–30 Hz) frequencies, compared to HC. Disrupted global connectivity in alpha frequencies with bilateral frontal cortices was associated with more severe clinical psychopathology (i.e., positive psychotic symptoms). Specifically, aberrant connectivity in beta frequencies between the left primary auditory cortex and cerebellum, was linked to greater hallucination severity in SZ. Disrupted connectivity in delta-theta frequencies between the medial frontal and left inferior frontal cortex was associated with impaired cognition.

**Discussion:**

The multivariate techniques employed in the present study highlight the importance of applying our source reconstruction techniques which leverage the high spatial localization abilities of MEG for estimating neural source activity using beamforming methods such as SAM (synthetic aperture morphometry) to reconstruct the source of brain activity, together with functional connectivity assessments, assayed with imaginary coherence metrics, to delineate how neurophysiological dysconnectivity in specific oscillatory frequencies between distinct regions underlie the cognitive and psychotic symptoms in SZ. The present findings employ powerful techniques in spatial and time-frequency domains to provide potential neural biomarkers underlying neuronal network dysconnectivity in SZ that will inform the development of innovations in future neuromodulation treatment development.

## Introduction

Schizophrenia is a devastating psychiatric disorder in which patients suffer from severe cognitive symptoms and psychotic symptoms of hallucinations and delusions. The term “Schizophrenia” was coined by Bleuler to suggest that psychotic symptoms result from the splitting of mental faculties. Indeed, recent accumulating evidence indicate that these cardinal cognitive and psychotic symptoms result from functional dysconnectivity (i.e., disrupted temporally correlated neural oscillatory frequency patterns) between cortical regions, resulting in impairments in neural communication (Hinkley et al., 2011; Liddle et al., 2016). Antipsychotic medications are not adequate with up to 40% of patients with schizophrenia (SZ) showing limited response (Lowe et al., 2018), thus compelling the need to understand the neurobiology underlying the functional decoupling that is thought to induce the debilitating cognitive and psychotic symptoms manifested in SZ.

A critical functional feature of neural oscillatory coupling between cortical regions is that their temporally correlated firing particularly at lower frequencies (<30 Hz) during resting-state represents the coordination, integration, and neurotransmission of signaling computation patterns between critical regions in frontal, temporal, and parietal cortices that are fundamental for healthy functioning (Calhoun et al., 2008; Hinkley et al., 2011; Hunt et al., 2017). This understanding of a fundamental neural network of connectivity underlying healthy functioning indicate that the regions that manifest aberrant connectivity in SZ, which would otherwise show, for example, reduced temporal correlations in healthy control participants (HC), would reveal how underlying dysfunctional amplified coupling between cortical regions in SZ induce a pathological heightened significance of irrelevant commonplace incidences, leading to cognitive distortions and positive psychotic symptoms of hallucinations in SZ (Kapur, 2003; Calhoun et al., 2008; Hinkley et al., 2011; Liddle et al., 2016; Hunt et al., 2017).

In the present study, we capitalize on the high spatiotemporal resolution of magnetoencephalography imaging (MEG) to record spontaneous neuronal activity during rest in SZ and HC. We focused on examining global changes in functional connectivity throughout the brain in delta/theta (2–8 Hz), alpha (8–12 Hz), and beta (12–30 Hz) frequency bands, rather than higher-frequencies which are sparsely reported at rest (Newson and Thiagarajan, 2018). Moreover, we focused on lower frequencies such as delta-theta, alpha, and beta band frequencies below 30 Hz, because they have shown to be dysfunctional in SZ, and hence we hypothesized these frequencies would reveal the most prominent differences in functional connectivity compared to HC (Canuet et al., 2011; Hinkley et al., 2011; Liddle et al., 2016; Hunt et al., 2017). Delta-theta, alpha, and beta oscillations play a critical role in integrative connectivity processes, making them ideal for examining changes in global functional coupling between cortical regions throughout the brain (Nunez et al., 2001; Hinkley et al., 2011; Liddle et al., 2016; Hunt et al., 2017). Alpha band oscillations represent an idling rhythm that are prevalent during rest across MEG, electroencephalogram (EEG) (Nunez, 1981), and functionally resting-state functional magnetic resonance imaging (fMRI) data (Mantini et al., 2007; Jann et al., 2009). We have previously shown that SZ reveal disrupted changes in global connectivity between cortical regions in alpha frequencies that predicted worsening psychotic symptoms (Hinkley et al., 2011). Delta-theta frequencies have also shown to be integral for mediating cortico-cortical communication that is necessary for cognition and are disrupted in SZ, impairing temporal communication between cortical regions (Guich et al., 1989; Hunt et al., 2017; Cao et al., 2022). Finally, prior research has shown that SZ, compared to HC, manifest amplified beta synchronization in response to irrelevant stimuli compared to relevant stimuli, which leads to the misplaced attribution of salience to irrelevant events, leading to hallucinations (Liddle et al., 2016). Collectively, these prior findings suggest that disruptions in delta-theta, alpha, and beta oscillations in SZ underlie aberrant perceptual/cognitive distortions, that lead to the cognitive and psychotic symptoms in SZ (Hinkley et al., 2011; Liddle et al., 2016; Hunt et al., 2017).

We also predicted that specific cortical fields in the frontal and temporal lobes would exhibit disrupted resting-state functional connectivity in SZ, consistent with compelling evidence revealing that SZ show disruptions in specific regions such as the medial prefontal cortex and primary auditory cortex, which underlie dysfunctional predictive coding mechanisms that blur the boundary between self-generated and external action outcomes (Ford et al., 2007, 2014; Subramaniam et al., 2012; Ford, 2016; Subramaniam, 2021). Normally in HC, the expected sensory outcomes of self-generated actions are well-predicted such as during speaking, for example, inducing suppression in the primary auditory cortex, compared to listening to external speech (Ford et al., 2007, 2014; Ford, 2016). Such suppression is thought to be the basis for the capacity to experience self-agency, which allows self-generated speech to be distinguished from externally derived speech (Ford and Mathalon, 2012; Chang et al., 2013). In other words in HC, self-generated (and therefore highly predictable) sounds give rise to suppressed responses, thus allowing speakers to pay better attention to sounds in the external environment (Ford and Mathalon, 2012; Chang et al., 2013), indicative of a primordial biological basis for self-agency that is essential for normal interactions with outside reality (Salomon and Starr, 1963; Poulet and Hedwig, 2006; Ford and Mathalon, 2012). By contrast, in SZ, exaggerated responses to self-generated actions (i.e., during speaking, for example) suggests patients may have noisier primary auditory cortical signals to begin with, making it more difficult to distinguish self-generated thoughts/speech from external speech (Ford et al., 2007, 2014; Ford, 2016). This auditory cortical signal is sent to higher order regions such as the mPFC that is considered to be critical for mediating higher-order cognitive processing underlying self-agency judgments (Korzyukov et al., 2017; Subramaniam et al., 2018, 2020; Subramaniam, 2021). We and others have provided consistent evidence from fMRI studies in which SZ show aberrant activity and connectivity within the medial prefrontal cortex that mediates the higher-order cognitive mechanisms required for making reliable predictions about the expected outcome of one’s own action that is necessary for self-agency (i.e., the awareness of being the agent of one’s own thoughts and actions) and for successful interactions with the outside world (Subramaniam et al., 2012, 2019, 2020; Kuhn and Gallinat, 2013; Robinson et al., 2016; Subramaniam, 2021).

Taking the above findings together, we specifically hypothesized that we would find functional disconnectivity in primary auditory cortices and mPFC, that would result from “noisier” computations that are delivered to mPFC from auditory cortex while patients were engaging in introspective internal thought processing at rest, which would lead to dysfunctional predictive coding mechanisms and hallucinations. This would be manifested by SZ showing exaggerated coupling in primary auditory cortex and mPFC with regions that would normally be suppressed in HC, distorting the boundary between internal thoughts and external speech, such that internal thoughts would be misattributed as external voices in the form of hallucinations (Kapur, 2003; Ford et al., 2007; Ford, 2016; Kort et al., 2017). As a result of the “noisier” computations that are delivered to mPFC from the auditory cortex that mediates dysfunctional predictive coding mechanisms (Ford and Mathalon, 2012; Ford et al., 2014; Ford, 2016; Mathalon et al., 2018), the mPFC would need to “work much harder” in SZ to mediate the higher-order cognitive mechanisms (i.e., attention, working-memory and executive functions) required for making computations based on prior experiences about how much to rely on the self-prediction signals about the expected outcome of one’s own actions, that is necessary for self-agency (Subramaniam et al., 2012, 2018, 2020; Subramaniam, 2021). To investigate whether abnormal oscillations in these specific medial prefrontal and primary auditory cortical sites were functionally related to pathological cognitive and psychotic symptoms, we examined functional connectivity between these specific sites as seed region-of-interest with voxels in the rest of the brain, to delineate how impaired neural oscillatory interactions in these regions related to psychopathology in SZ. Our objective was to examine the specific sites that show dysfunctional neural oscillatory coupling in SZ and to examine how decoupling relates to pathophysiological cognitive and clinical symptoms, with the aim of informing the development of future novel neuromodulation-based treatment approaches.

## Materials and methods

### Participants and procedures

This study represents the baseline MEG resting-state portion of a NIMH-funded R01 (R01MH122897) study in schizophrenia to KS. Twenty-one volunteer SZ participants and 21 HC participants, matched at a group level on age and gender, completed this MEG study at the University of California San Francisco (UCSF) (see Table 1). SZ and HC participants had volunteered to participate from our clinicaltrial.gov site (NCT04807530) or had volunteered to participate from our previous clinical studies in schizophrenia if they had consented to be contacted for future studies. Inclusion criteria were Axis I diagnosis of schizophrenia, assessed with the [Structured Clinical Interview for DSM-V (SCID)] or, for HC, no Axis I or Axis II psychiatric disorder (SCID–Non-patient edition), no substance dependence or abuse, age between 18 and 60 years, and English as first language. All subjects gave written informed consent for this protocol approved by the Institutional Review Board (IRB) at UCSF, and then completed cognitive and clinical assessments and MEG imaging.

**TABLE 1:** Demographics, clinical symptoms, and antipsychotic medication (Mean, SD) of schizophrenia patients (SZ) and healthy comparison subjects (HC).

	HC	SZ	*P*-value
	(*N* = 21)	(*N* = 21)	
Age	42 (11.6)	45 (8.5)	0.3
Gender	15M, 6F	16M, 5F	0.7
Illness duration (y)	N/A	23 (13.6)	N/A
Positive symptoms	N/A	16 (5.7)	N/A
Negative symptoms	N/A	17 (4.9)	N/A
Chlorpromazine (CPZ) equivalents	N/A	297 (148)	N/A

### Clinical and neurocognitive assessments

Schizophrenia subjects received clinical and cognitive assessments. Clinical symptoms were assayed with the Positive and Negative Syndrome Scale (PANSS) (Kay et al., 1987). Cognition was assessed with the MATRICS Consensus Cognitive Battery (MCCB) (Nuechterlein et al., 2008), which included assessments of attention (Continuous Performance Task-Identical Pairs Test); working memory (Letter-Number Span Test); and executive functioning (NAB Mazes Test).

### Data acquisition

Each participant underwent 4 min of continuous resting-state recording inside a magnetically shielded room with a 275-channel whole-head MEG system (MEG International Services Ltd., Coquitlam, British Columbia, Canada) consisting of 275 axial gradiometers. This study protocol required participants to be in a supine position with eyes closed (sampling rate = 1.2 kHz).

To provide anatomical head models for MEG analysis, a high-resolution 3D T1-weighted whole-brain magnetic resonance imaging (MRI) was acquired for each subject using a 3T Siemens scanner. For each subject, the outline of the brain on the structural scans was extracted, and the segmented brain was treated as a volume conductor model for the source reconstruction described below. Three fiducial coils (nasion, left and right preauricular points) were placed to localize the position of the head relative to the MEG sensor array. Co-registration of the MEG data with each individual’s structural anatomical MRI was performed based on the nasion and left and right preauricular fiducial coil positions.

### Data analysis

The Nutmeg software suite^1^ was used to compute MEG source reconstruction and functional connectivity metrics. We capitalize on the high spatiotemporal resolution of MEG to assay time-frequency neural oscillatory connectivity in different frequency bands. A 60-s artifact-free segment of the MEG data was chosen from the 4 min dataset. First, artifact detection was performed qualitatively through visual inspection of the sensor data with only trials without excessive scatter (signal amplitude > 10 pT) due to eyeblink, saccades, head movement, or electromyograph noise were selected for data analysis. Then, the MEG sensor data was filtered with a phase-preserving bandpass filter (fourth-order Butterworth; 1–55 Hz bandpass). We implemented beamforming methods such as SAM (synthetic aperture morphometry) to reconstruct neural source activity from the spatio-temporal patterns in sensor data. SAM estimates the timecourse of neural activity at every location from each subject’s anatomical MRI, while suppressing noise from activity in other locations. At each voxel, this technique offers an amplitude estimate derived through a linear combination of a spatial weights with the MEG sensor data. Through creating a multi-sphere head model based on an obtained head shape from the structural MRI of each subject, tomographic reconstructions of the data were generated for each individual subject. A whole brain VOI for lead field calculation (grid size 8 mm) was automatically created by means of a back-transformation of all the points within a spatially normalized MRI which only corresponded to locations within the brain, excluded non-cerebral foci.

Given that the magnetic field generated by one neural source is picked up not only by the closest sensor but also other near sensors with a zero time-lag, we combined SAM beamforming with functional connectivity techniques such as imaginary coherence (IC) to overcome this issue that results from neighboring sensors containing redundant signal. IC isolates non-zero time lag interactions from the source-reconstructed SAM data to cancel out the redundant zero-time lag cross-talk between sensors. In this way, IC efficiently samples source time series communication, independent of the spatial filter applied, providing a precise powerful technique for examining functionally connected resting-state networks. For each subject, the time frequency analyses was run on three frequency ranges (2–7 Hz for delta-theta, 8–12 Hz for alpha, and 12–30 Hz for beta). For each frequency band, bivariate IC values between two voxels X and Y were calculated as follows:

$$I\,C_{X\,Y}=\left|l\,m\,\frac{\sum_{K=1}^{K}X_{K}\,Y_{K}}{\sqrt{\sum_{K=1}^{K}{\left|X_{K}\right|}^{2}\,\sum_{K=1}^{K}{\left|Y_{K}\right|}^{2}}}\right|$$

Both global connectivity and seeded connectivity were computed in the analyses. Global connectivity at each voxel was computed by averaging the voxel’s Fisher’s *z*-transformed IC values with all the other voxels. Seeded connectivity was computed as the connectivity to a seed ROI, by averaging across *z*-transformed IC values between the peak voxel in the seed region and all other voxels. Based on our *a priori* hypotheses, we selected to use the mPFC and primary auditory cortex as our seed ROIs.

Spatial normalization was applied to the T1-weighted MRIs (5 mm; SPM12^2^) and then the derived transformation matrix from the normalization was applied to the global connectivity maps for each individual subject. Between group contrasts of SZ vs. HC were conducted with a 2-sample *t* test. All second-level group analyses were corrected using False Discovery Rate (FDR) for multiple comparisons (FDR *p* < 0.05). We used Pearson’s correlations to examine the strength of associations between connectivity values with cognition and clinical symptom scores.

## Results

### Global functional connectivity: Differences between HC and SZ

**FIGURE 1:**
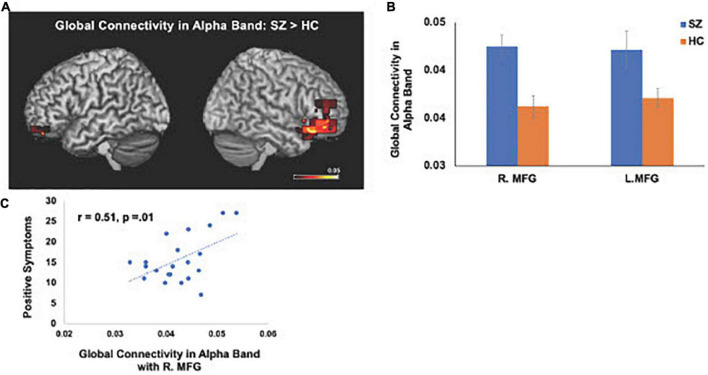
Between-group comparisons reveal that compared to HC, SZ show amplified global connectivity in alpha frequencies with bilateral MFG **(A,B)** which predicted more severe positive symptoms **(C)** (FDR, *p* < 0.05).

**FIGURE 2:**
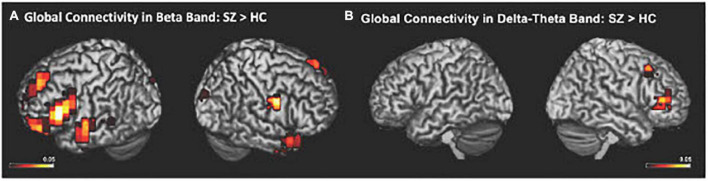
Between-group comparisons reveal that compared to HC, SZ showed amplified global connectivity in beta **(A)** and delta-theta frequencies **(B)** (FDR, *p* < 0.05).

The IC metrics provide computations of the global functional connectivity at each voxel between that region and the rest of the brain. In a separate cohort of HC participants, test-retest reliability of global connectivity maps was evaluated by calculating the intra-class correlation coefficient (ICC). Good test-retest reliability was confirmed in the global connectivity maps for both within session (ICC = 0.61) and between baseline scans and follow-up sessions several weeks later (ICC = 0.64) (Hinkley et al., 2011). All second-level group analyses were corrected using False Discovery Rate (FDR) for multiple comparisons (FDR *p* < 0.05). In delta-theta, alpha, and beta frequencies, we found similar patterns of global connectivity across functionally critical brain regions of parietal, temporal, occipital, and frontal cortices in both HC and SZ groups. However, we also found significant differences in global connectivity between the HC and SZ groups in alpha, beta, and delta-theta bands (FDR *p* < 0.05) (see Table 2). Specifically, increased global connectivity in alpha frequencies in SZ, compared to HC, was restricted to bilateral middle frontal gyri (MFG) (FDR, *p* < 0.05). Increased alpha connectivity with the R.MFG predicted more severe positive symptoms (Figure 1). We also found increased global connectivity in beta frequencies in SZ compared to HC, in several regions, including left superior/inferior frontal gyrus (L.S/IFG), bilateral superior temporal gyri, and right precentral gyrus (R.PCG) (FDR, *p* < 0.05) (Figure 2A). Finally, increased global connectivity was found in delta-theta frequencies in R.MFG (FDR, *p* < 0.05) (Figure 2B).

**TABLE 2:** Group differences in global connectivity in alpha, beta, and delta-theta frequencies.

Region	Abbrev	Hemisphere	BA	*x*	*y*	*z*	*p*	*T*
***Group comparison in alpha band***								
Middle frontal gyrus	MFG	L	11	−31	41	−15	0.026	2.32
Middle frontal gyrus	MFG	R	11	24	37	−12	0.0005	3.81
***Group comparison in beta band***								
Superior frontal gyrus	SFG	L	9	−17	43	37	0.001	3.71
Superior temporal gyrus	STG	L	22	−46	−11	−7	0.003	3.13
Inferior frontal gyrus	IFG	L	45	−55	20	23	0.0005	3.81
Superior temporal gyrus	STG	R	38	34	6	−24	0.002	3.26
Precentral gyrus	PCG	R	6	47	−3	33	0.002	3.40
***Group comparison in delta-theta band***								
Middle frontal gyrus	MFG	R	11	32	37	−1	0.001	3.51

### Seeded functional connectivity: Differences between HC and SZ

**FIGURE 3:**
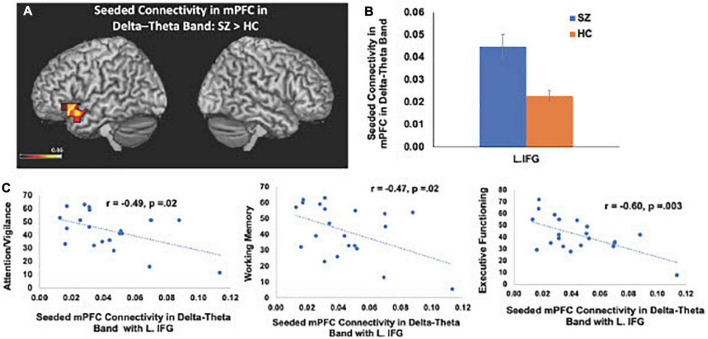
Between-group comparisons reveal that compared to HC, SZ show amplified mPFC seeded connectivity with L.IFG in delta-theta frequencies (FDR, *p* < 0.05) **(A,B)**, which predicted poorer cognition **(C)**.

Consistent with our *a priori* hypotheses, we found significant differences in seeded connectivity between the HC and SZ groups in mPFC and primary auditory cortices (see Table 3). Specifically, we found that the SZ group showed increased mPFC connectivity in delta-theta frequencies with left inferior frontal gyrus, extending to superior temporal gyrus (L.IFG/L.STG) (FDR, *p* < 0.05) (Figures 3A, B). Importantly, amplified mPFC connectivity in delta-theta frequencies with L.IFG predicted poorer cognition (i.e., poorer attention, working memory, and executive functioning) (Figure 3C).

**TABLE 3:** Group differences in seeded connectivity in mPFC and left primary auditory cortex.

Region	Abbrev	Hemisphere	BA	*x*	*y*	*z*	*p*	*T*
***Group comparison in mPFC seeded connectivity in delta-theta band***								
Inferior frontal gyrus	IFG	L	47	−32	21	−17	0.001	3.57
***Group comparison in left primary auditory cortex seeded connectivity in alpha band***								
Cerebellum	CBM	R		40	−83	−33	0.004	3.01
***Group comparison in left primary auditory cortex seeded connectivity in beta band***								
Cerebellum	CBM	L		−8	−51	−58	0.0005	3.82
Precentral gyrus	PCG	L	6	−57	3	40	0.003	3.13
Cerebellum	CBM	R		48	−75	−49	0.003	3.14

**FIGURE 4:**
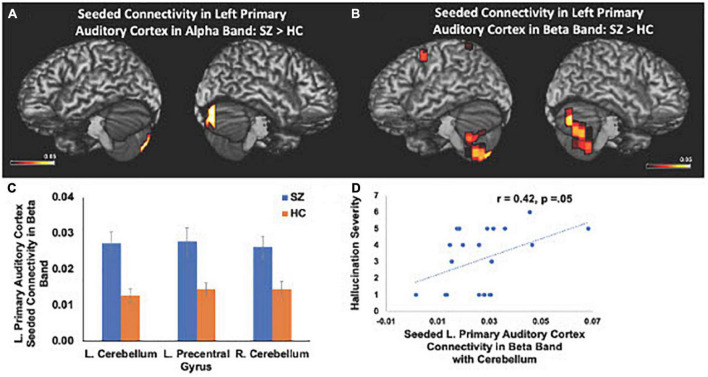
Between-group comparisons reveal that compared to HC, SZ show amplified left primary auditory cortex seeded connectivity with the cerebellum in both alpha **(A)** and beta frequencies **(B,C)** (FDR, *p* < 0.05), which predicted more severe hallucinations **(D)**.

We also found significant increases in left primary auditory cortex seeded connectivity in both alpha and beta frequencies in SZ, compared to the HC group (FDR, *p* < 0.05). In particular, we found amplified connectivity in both alpha and beta frequencies between the left primary auditory cortex with bilateral cerebelli (FDR, *p* < 0.05) (Figures 4A–C). Finally, heightened connectivity between left primary auditory cortex with the left cerebellum in beta frequencies predicted more severe hallucination severity (Figure 4D). We did not find any associations between either global or seeded IC metrics with age, gender, illness duration, negative symptoms or antipsychotic medication (all *p*’s > 0.05).

## Discussion

We apply MEG source reconstruction beamforming techniques using SAM to leverage the high spatial localization abilities of MEG, together with functional connectivity, assayed with imaginary coherence metrics, to present direct evidence for amplified functional connectivity in distinct frequency bands between specific cortical regions in SZ, compared to the HC group. We found that aberrant delta-theta, alpha, and beta frequencies predicted distinct phenotypic positive psychotic and cognitive symptoms in SZ. Greater global connectivity was found in alpha frequencies with R. MFG, which predicted more severe positive psychotic symptoms in SZ (Figure 1). SZ also manifested increased global connectivity between frontal and temporal regions in beta and delta-theta frequencies (Figure 2). Amplified mPFC connectivity in delta-theta frequencies with L.IFG/L.STG specifically predicted more impaired cognition. Collectively, the present findings are consistent with prior fMRI studies revealing that SZ manifest global dysfunctional connectivity particularly with frontal and temporal regions which correlated with cognitive impairments and psychotic symptoms (MacDonald et al., 2005; Wolf et al., 2011).

Here, we replicate and extend these prior studies by applying MEG to delineate the precise oscillatory frequencies whose aberrations in connectivity between distinct cortical regions in SZ underlie cognitive and psychotic symptoms. Specifically, we found that amplified mPFC connectivity with L.IFG in delta-theta frequencies in SZ predicted greater cognitive impairments in attention, working memory and executive functioning (Figure 3). Additionally, aberrant global connectivity with R.MFG in alpha frequencies predicted greater overall positive symptoms, while aberrant connectivity in primary auditory cortex with cerebellum in beta frequencies in SZ specifically predicted more severe hallucination symptoms (Figure 4). These findings suggest that the aberrant resting-state connectivity in alpha, beta and delta-theta bands between these critical regions reflects potential neural biomarkers that underlie the cognitive and psychotic symptoms in SZ, which are likely to provide useful treatment targets through behavioral and neuromodulation interventions, such as transcranial magnetic stimulation therapies.

Alpha oscillations dominate during rest when subjects are relaxed, and represent a stable rhythm, that underlie long-range cortico-cortical brain interactions (Nunez, 1974; von Stein and Sarnthein, 2000; Nunez et al., 2001; Nikulin and Brismar, 2005). Our findings revealing aberrant alpha connectivity in SZ, which predicted more severe positive symptoms, are consistent with our previous MEG work showing global compromised alpha oscillatory connectivity patterns in SZ (Hinkley et al., 2011). Collectively, these convergent findings between the present findings and our prior work suggest that frontal regions within the MFG play a prominent role in long-range connectivity during resting-states, but show amplified global connectivity with MFG in SZ, suggesting that amplified long-range MFG connections in SZ may contribute to formation of excessive disparate connections that are effectively random (Rubinov et al., 2009; Rubinov and Bassett, 2011). Such a randomized network structure would potentiate the strengthening of unnecessary connections, leading to abnormal synchrony in spatiotemporal coupling between regions that are normally segregated in HC, but show enhanced connectivity in SZ (Rubinov et al., 2009), inducing impairments in filtering out irrelevant from relevant salient stimuli in SZ, leading to positive psychotic symptoms (e.g., hallucinations in which SZ appear to hear auditory stimuli such as voices when there is no external auditory stimuli).

The present findings also provide an underlying neural framework that supports Kapur’s theory (Kapur, 2003) that psychosis arises from aberrant salience processing, in which SZ attribute amplified attention and salience for irrelevant stimuli, leading to hallucinations in SZ. Beta oscillatory frequencies are considered to be fundamental for mediating salience to relevant information (Liddle et al., 2016). Liddle et al. (2016) extended Kapur’s theory of abnormal salience in SZ to show that abnormal beta rhythms underlied hallucinations in SZ. In particular, Liddle et al. (2016) designed a salience detection MEG task in which participants were required to distinguish relevant stimuli from irrelevant stimuli in order to delineate the neural mechanisms underlying salience detection in HC and SZ (Liddle et al., 2016). They found that SZ, compared to HC, showed significantly larger beta oscillatory activity for irrelevant stimuli. Consistent with these prior findings, in the present study, we found that SZ showed increased global connectivity in beta frequencies with frontal and temporal cortices. In particular, we found functional disconnectivity in the left primary auditory cortex, which showed exaggerated coupling in beta oscillatory frequencies with the cerebellum that is also known to mediate self-agency (Blakemore et al., 2001; Synofzik et al., 2008; Crivelli and Balconi, 2017; Welniarz et al., 2021), which was associated with more severe hallucinations in SZ.

Both primary auditory cortices and cerebellum are thought to compute a prediction error in signaling the discrepancy between the predicted and actual sensory outcome of one’s own actions (Blakemore et al., 2001; Ford et al., 2007; Synofzik et al., 2008; Ford, 2016; Crivelli and Balconi, 2017; Welniarz et al., 2021). Across the animal kingdom, self-produced actions are well-predicted, and thus, we experience self-agency when the sensory outcome of self-generated actions minimally deviates from the expected sensory outcome and is suppressed, compared to external-action outcomes (Salomon and Starr, 1963; Poulet and Hedwig, 2006; Ford et al., 2014; Ford, 2016; Korzyukov et al., 2017; Subramaniam et al., 2018). The present findings revealing abnormal amplified connectivity between primary auditory cortex and cerebellum, which predicted more severe hallucinations, extend prior reports showing both abnormal primary auditory cortical and cerebellar responses to self-generated speech sounds, indicating dysfunctional predictive mechanisms that lead to an aberrant sense of agency (Ford et al., 2007, 2014; Ford, 2016; Moberget and Ivry, 2019). Collectively, the present results and prior studies indicate that SZ show heightened responses to self-generated actions, which are normally suppressed in HC, leading to the misattribution of self-generated sounds as external voices in the form of hallucinations (Ford et al., 2007, 2014; Ford, 2016; Moberget and Ivry, 2019).

It must also be noted that with traditional 10–20 EEG and MEG systems, there is lower signal-to-noise ratio in the cerebellum as a result of the larger distance between the cerebellum and the sensors. However, with our 275-channel whole-head MEG system, we are able to provide good cerebellar coverage by placing sensors as close as possible to the cerebellum. Further, using multiple layers of sensors provide better separation of signal between the cerebral cortex and signal in the cerebellum such as with our MEG CTF system (MEG International Services Ltd., Coquitlam, British Columbia, Canada). Finally, the implementation of a multi-sphere head model and time-frequency beamforming analyses for source localization which we have implemented here, have been demonstrated to be an effective method for localizing signal from sources in the cerebellum, as also shown by several previous MEG reports listed in Table 1 in Andersen et al. (2020) and in other MEG papers (Hämäläinen et al., 1993; Brookes et al., 2011). For example, Brookes et al. (2011) specifically used beamforming to provide evidence for consistent overlap between MEG resting-state networks with fMRI, and showed evidence of cerebellar resting-state activation in beta band, as also confirmed by our findings here.

We have also consistently shown that SZ reveal aberrant activity and connectivity within the mPFC that mediates the higher-order cognitive mechanisms required for making reliable predictions about the expected outcome of one’s own action that is necessary for self-agency (Subramaniam et al., 2012, 2019, 2020; Kuhn and Gallinat, 2013; Robinson et al., 2016; Subramaniam, 2021). Here, we extend these prior studies by using MEG to delineate the specific frequencies that underlie mPFC functional dysconnectivity. We specifically found amplified connectivity in delta-theta frequencies between the LIFG (Broca’s Area), extending to L.STG region. The present findings are consistent with prior fMRI functional connectivity studies showing amplified fMRI connectivity with this L.IFG/L.STG region in hallucinating SZ, compared to HC (Hoffman and Hampson, 2011; Hoffman et al., 2011). Normally in HC, the L.IFG region is activated when listening to external speech as well as prior to speech onset, as this region is thought to mediate the higher-order multicomponent cognitive preparation (involving attention, working-memory and executive functioning components) that is necessary for integrating semantic and syntactic information to produce comprehensible speech (Flinker et al., 2015). By contrast, the amplified coupling to L.IFG/L.STG observed in SZ in the absence of external speech, suggests the misattribution of inner thoughts and speech as external voices, manifested as verbal hallucinations (Hoffman and Hampson, 2011; Hoffman et al., 2011). Our findings show that even when subjects are at rest, in the absence of external speech, we find amplified mPFC connectivity in delta/theta frequencies with L.IFG/L.STG, which was associated with greater impairments cognitive process of attention, working-memory and executive functions. The present findings converge well with prior studies showing amplified delta/theta connectivity, contributing to cognitive impairments in SZ (Guich et al., 1989; Cao et al., 2022). Taken these findings together, the present results suggest that the amplified coupling between mPFC and L.IFG/LSTG likely contribute to the higher-order multicomponent cognitive impairments that underlie self-agency (Subramaniam et al., 2012, 2018, 2020; Subramaniam, 2021).

There are some limitations of the present study. Firstly, the present findings are applicable to chronically ill SZ, and therefore, we do not know whether these findings would extend to ultra-high risk or recent onset SZ. Secondly, this study only examined functional connectivity at rest and did not examine neural functional connectivity differences between HC and SZ during task performance. Examining functional connectivity during task performance would provide further insight into the neural mechanisms underlying cognitive dysfunction in schizophrenia. We also did not analyze very low frequencies (e.g., 1–2 Hz frequencies) because enclosing the MEG system within a shielded room for reduction of environmental noise is not effective at these low-frequencies (Vrba and Robinson, 2001). Additionally, at these very low frequencies, MEG brain signals are also confounded by body oscillations such as breathing and heart rate. Furthermore, typical filters do not allow for isolated examination of these frequencies without leakage effects from lower and higher frequencies. We did not also analyze frequencies above 30 Hz for resting-state networks because we and others have found that gamma power shows low test-retest reliability using intraclass correlation coefficients (ICC) for resting-state analyses (Martin-Buro et al., 2016). Finally, while MEG resting-state scans are non-invasive and can be acquired rapidly (4 mts duration), MEG is not widely available, limiting a more extensive use of this technology; however, implementing similar pipelines for more widely available EEG data for source localization resting-state analyses would facilitate fast and easy-to-implement valuable information regarding the neural aberrations underlying cognitive and psychotic symptoms that we foresee would be the first step to delineating neuromodulation treatments for implementation and transition to the clinic.

In summary, most previous studies have focused on univariate functional connectivity analyses in SZ, focusing on the spatial domain (e.g., in fMRI studies) (Hoffman and Hampson, 2011; Hoffman et al., 2011) or in the case of EEG, studies have been limited to spatial restrictions of EEG-sensor based analyses (Ford et al., 2001, 2014; Ford, 2016) that prevent us from combining both high-resolution spatial and time-frequency information to examine precisely which specific regions in distinct frequency spectra contribute to psychopathology in SZ. In the present study, we employ multivariate techniques which highlight the importance of applying our source reconstruction techniques, which capitalize on the high spatial localization abilities of MEG, together with applying global and seed-based functional connectivity, assayed with imaginary coherence metrics in different frequency spectra, to demonstrate precisely how neurophysiological dysconnectivity in specific oscillatory frequencies between distinct regions underlie the cognitive and psychotic symptoms in SZ. The present findings employ robust algorithms in spatial and time-frequency domains to provide potential neural biomarkers underlying neuronal network dysconnectivity in SZ for predicting distinct phenotypic cognitive and psychotic symptoms that will inform the development of innovations in future neuromodulation treatment development.

## Data availability statement

The original contributions presented in this study are included in the article/supplementary material, further inquiries can be directed to the corresponding author.

## Ethics statement

The studies involving human participants were reviewed and approved by Institutional Review Board at UCSF. The patients/participants provided their written informed consent to participate in this study.

## Author contributions

YJ acquired and analyzed all the data and helped to write the manuscript. NJ helped with subject recruitment and acquisition of the data. LH provided advice on the analyses of the data. SN provided advice on analyses and interpretation of the data. KS provided supervision on acquisition and analyses of the data and wrote, edited, and submitted the manuscript. All authors contributed to the article and approved the submitted version.

## Footnotes

1
http://nutmeg.berkeley.edu

2
http://www.fil.ion.ucl.ac.uk/spm/
